# Comparison of ^18^FDG PET/PET-CT and bone scintigraphy for detecting bone metastases in patients with nasopharyngeal cancer: a meta-analysis

**DOI:** 10.18632/oncotarget.20026

**Published:** 2017-08-07

**Authors:** Chuanhui Xu, Ruiming Zhang, Huanlei Zhang, Zhenyan Zhang

**Affiliations:** ^1^ Department of Radiology, Yidu Central Hospital of Weifang, Qingzhou 262500, Shandong, PR China

**Keywords:** PET, bone scintigraphy, nasopharyngeal cancer, staging, bone metastases

## Abstract

**Objective:**

We performed a meta-analysis to compare the diagnostic efficacy of ^18^FDG PET/PET-CT and bone scintigraphy (BS) for diagnosing bone metastatic cancers in nasopharyngeal cancer patients.

**Results:**

6 studies (1238 patients) fulfilled the inclusion criteria. The pooled sensitivities for ^18^FDG PET/PET-CT and BS were 0.81 (95% confidence interval [CI] = 0.70 to 0.98) and 0.39 (95% CI = 0.26 to 0.54), specificities were 0.99 (95% CI = 0.98 to 0.99) and 0.98 (95% CI = 0.96 to 0.99), and the areas under curve were 0.98 (95% CI = 0.97 to 0.99) and 0.84 (95% CI = 0.81 to 0.87).

**Materials and Methods:**

Several databases were searched for all available articles. We calculated the sensitivities, specificities, diagnostic odds ratios, likelihood ratios, and area under summary receiver operating characteristic curves for ^18^FDG PET/PET-CT and BS, respectively.

**Conclusions:**

^18^FDG PET/PET-CT is superior to BS for diagnosing bone metastases in nasopharyngeal cancer patients.^18^FDG PET/PET-CT may enhance the diagnosis of bone metastases and provide more accurate information for the optimal management of nasopharyngeal cancer.

## INTRODUCTION

Bone is a very common site of distant metastases in nasopharyngeal cancer patients [1–3]. Bone metastasis accounts for approximately 10–16% of all patients with nasopharyngeal cancer and 60–80% of patients with distant metastasis at initial diagnosis [1–3]. The selected treatment strategies, such as radiotherapy, chemotherapy, and targeted therapy is mainly dependent on the TNM staging. If bone metastasis is found, the treatment strategies may change significantly. The accurate assessment of bone metastasis is necessary for M staging and the selection of optimal treatment.

Various techniques of diagnostic imagings, such as bone scintigraphy (BS), positron emission tomography (PET), PET/computed tomography (CT), and magnetic resonance imaging are widely used for the assessment of bone metastasis. ^99m^Tc-phosphonate BS is most widely used to assess bone metastasis for many decades because of its ability to evaluate the entire skeleton at a relatively low cost. BS relies on the osteoblastic response to bone destruction by tumor cells [4]. But the high false positive rate of BS may be caused by some benign processes (osteoarthritis, fractures, degenerative changes, etc) [5]. As a functional imaging modality,^18^FDG PET can detect potential tumor activity and facilitate earlier detection of bone metastatic lesions. The introduction of PET-CT has combined the functional imaging of PET with the anatomic imaging of CT into a single examination. Several studies have validated the potential value of ^18^FDG PET/PET-CT and PBS for the assessment of bone metastases in nasopharyngeal cancer [1–3, 6–8]. However, the findings of ^18^FDG PET/PET-CT and BS are variable or incongruent. Therefore, we undertook a meta-analysis to compare the diagnostic efficacy of ^18^FDG PET/PET-CT and PBS in detecting bone metastases of nasopharyngeal cancer patients.

## RESULTS

### Study selection and description

**Figure 1:**
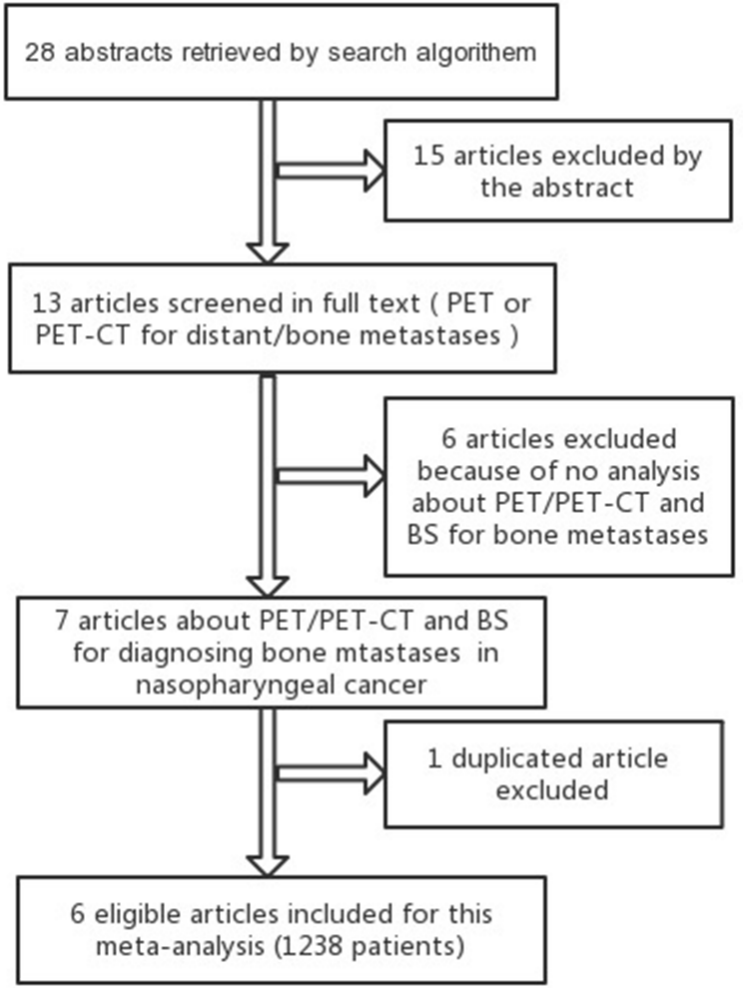
Shows the flow chart of search for eligible studies

The flow chart of search for eligible studies was showed in Figure 1. After independent review, 7 articles dealing with the comparison of ^18^FDG PET/PET-CT and BS for detecting bone metastases of nasopharyngeal cancer patients were eligible for this meta-analysis. Of seven articles, one article [9] was excluded because the data was already reported in an included article [1]. Consequently, 6 articles [1–3, 6–8] were included in this meta-analysis (Figure 1). A total of 1238 patients were analyzed for the diagnostic efficacy of ^18^FDG PET/PET-CT and BS (Table 1). In five articles (83.3%), the study design was prospective.

**Table 1:** The clinical characteristics of all included studies

Study	Country	No. of patients	Design	Type of staging	Age(y)	PET/PET-CT technique			BS technique		Internal time	Follow-up time
						Dose	CE-CT	Uptake time	Dose	Delay time		
Chan [8], 2006	Taiwan	131	Prosp	RS	unclear	370 MBq	No	≥ 40 min	925 MBq	3-4h	14 d	≥ 6 m
Liu [1], 2007	Taiwan	300	Prosp	IS	50.5	370MBq	No	≥ 40 min	925 MBq	3-4h	14 d	≥ 6 m
Chua [6], 2009	Singapore	78	Prosp	IS	NR	370 MBq	No	60 min	740 MBq	3h	14 d	≥ 12m
Ng [2], 2009	Taiwan	111	Prosp	IS	24-83	370 MBq	No	50-70 min	925 MBq	3-4h	10 d	≥ 12 m
Tang [3], 2013	China	583	Prosp	IS	46(mean)	5.55 MBq/kg	No	45-60 min	NR	NR	14 d	≥ 12 m
Yang [7], 2014	China	35	Retro	RS+IS	21-76	7.4 MBq/kg	No	60 min	925 MBq	2-4h	7 d	≥ 6 m

#IS = Initial Staging, RS = Restaging, Prosp = Prospective, Retro = Retrospective, NR = Not Reported, CE-CT = Contrast Enhanced-Computed Tomography.

### Study quality

Quality assessment of all included studies is shown in Table 2. Overall, the quality of the included studies was satisfactory. For all six studies, the results of ^18^FDG PET/PET-CT and BS was interpreted without any knowledge of the reference standard. But the reference standard wasn't executed without any knowledge of the results of ^18^FDG PET/PET-CT and BS in all included studies.

**Table 2:** QUADAS-2 results for all included studies

Studies	Risk of bias				Applicability concerns		
	Patient selection	Index test	Reference standard	Flow and timing	Patient selection	Index test	Reference standard
Chan [8], 2006	HR	LR	HR	LR	HR	LR	LR
Liu [1], 2007	LR	LR	HR	LR	LR	LR	LR
Chua [6], 2009	LR	LR	HR	LR	LR	LR	LR
Ng [2], 2009	LR	LR	HR	LR	LR	LR	LR
Tang [3], 2013	LR	LR	HR	LR	LR	LR	LR
Yang [7], 2014	HR	LR	HR	LR	HR	LR	LR

#HR = high risk, LR = high risk.

### Accuracy of ^18^F-FDG PET/PET-CT and BS

#### All included studies

**Figure 2:**
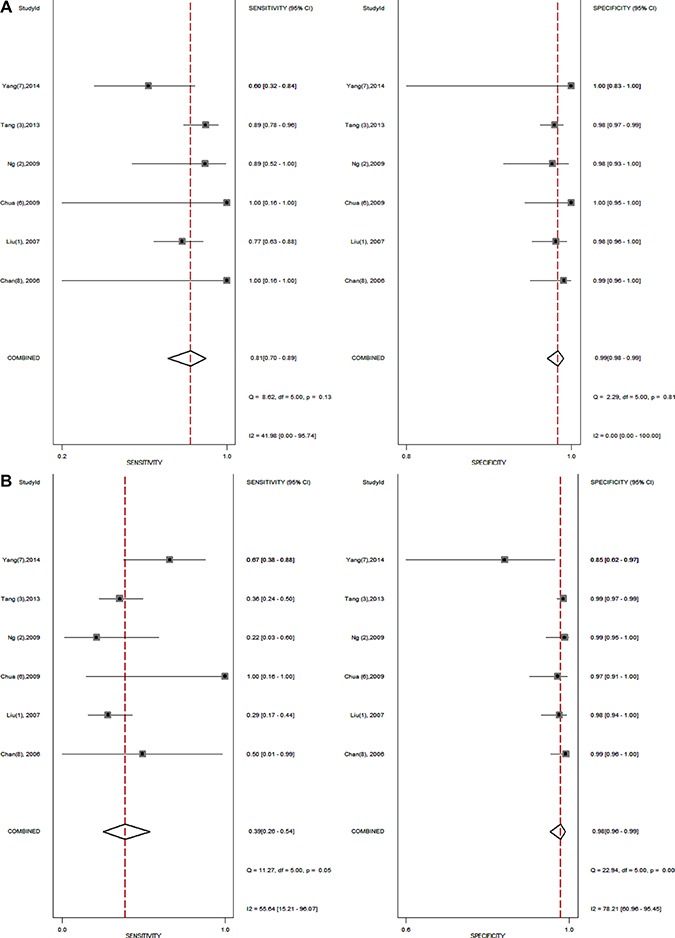
(**A**–**B**) shows the forest plot of sensitivity and specificity for ^18^FDG PET/PET-CT (**A**) and bone scintigraphy (**B**) from all 6 studies.

The forest plots of sensitivity and specificity for ^18^FDG PET/PET-CT and BS from all 6 studies (1238 patients) were shown in Figure 2A, 2B. When considering all 6 studies (1238 patients), the pooled sensitivity, specificity, DOR, PLR and NLR of ^18^FDG PET/PET-CT were 0.81 (95% confidence interval [CI] = 0.70 to 0.98), 0.99 (95% CI = 0.98 to 0.99), 312 (95% CI = 144 to 676), 58.6 (95% CI = 33.5 to 102.7) and 0.19 (95% CI = 0.11 to 0.31), respectively, and of BS were 0.39 (95% CI = 0.26 to 0.54), 0.98 (95% CI = 0.96 to 0.99), 32 (95% CI = 16 to 64), 19.9 (95% CI = 10.3 to 38.7) and 0.62 (95% CI = 0.49 to 0.78), respectively (Table 3).

**Table 3:** Accuracy of 18FDG PET-CT and bone scan for detection of bone metastases in nasopharyngeal cancer patients

Imaging Modalites	No. of Studies (No. of Patients)	Sensitivity (95% CI)	specificity(95% CI)	DOR (95% CI)	PLR (95% CI)	NLR (95% CI)
**All Studies**						
^18^FDG PET/PET-CT	6 (1238)	0.81 (0.70–0.89)	0.99 (0.98–0.99)	312 (144–676)	58.6 (33.5–102.7)	0.19 (0.11–0.31)
Bone Scan	6 (1238)	0.39 (0.26–0.54)	0.98 (0.96–0.99)	32 (16–64)	19.9 (10.3–38.7)	0.62 (0.49–0.78)
**Prospective Studies**						
^18^FDG PET/PET-CT	5 (1203)	0.85 (0.75–0.91)	0.99 (0.98–0.99)	373 (173–806)	57.7 (35.1–94.8)	0.15 (0.09–0.26)
Bone Scan	5 (1203)	0.34 (0.26–0.43)	0.98 (0.98–0.99)	34 (18–66)	23.2 (13.0–41.5)	0.67 (0.59–0.77)
**PET-CT system**						
^18^FDG PET-CT	4 (807)	0.83 (0.65–0.99)	0.99 (0.97–0.99)	351 (115–1077)	61.6 (24.8–153.1)	0.18 (0.08–0.38)
Bone Scan	4 (807)	0.46 (0.28–0.65)	0.98 (0.93–0.99)	34 (14–78)	18.7 (7.7–45.2)	0.56 (0.39–0.79)
**Initial Staging**						
^18^FDG PET/PET-CT	4 (1072)	0.84 (0.75–0.91)	0.98 (0.97–0.99)	342 (158–740)	54.0 (32.3–90.3)	0.16 (0.10–0.26)
Bone Scan	4 (1072)	0.33 (0.25–0.42)	0.98 (0.97–0.99)	32 (16–62)	21.5 (11.8–39.1)	0.68 (0.59–0.77)

#### Prospective studies

When considering 5 prospective studies (1203 patients), the pooled sensitivity, specificity, DOR, PLR and NLR of ^18^FDG PET/PET-CT were 0.85 (95% CI = 0.75 to 0.91), 0.99 (95% CI = 0.98 to 0.99), 373 (95% CI = 173 to 806), 57.7 (95% CI = 35.1 to 94.8) and 0.15 (95% CI = 0.09 to 0.26), respectively, and of BS were 0.34 (95% CI = 0.26 to 0.43), 0.98 (95% CI = 0.98 to 0.99), 34 (95% CI = 18 to 66), 23.2 (95% CI = 13.0 to 41.5) and 0.67 (95% CI = 0.59 to 0.77), respectively (Table 3).

#### PET-CT system

When considering 4 studies with PET-CT system (807 patients), the pooled sensitivity, specificity, DOR, PLR and NLR of ^18^FDG PET-CT were 0.83 (95% CI = 0.65 to 0.99), 0.99 (95% CI = 0.97 to 0.99), 351 (95% CI = 115 to 1077), 61.6 (95% CI = 24.8 to 153.1) and 0.18 (95% CI = 0.08 to 0.38), respectively, and of BS were 0.46 (95% CI = 0.28 to 0.65), 0.98 (95% CI = 0.93 to 0.99), 34 (95% CI = 14 to 78), 18.7 (95% CI = 7.7 to 45.2) and 0.56 (95% CI = 0.39 to 0.79), respectively (Table 3).

#### Initial staging

When considering 4 studies at initial staging (1072 patients), the pooled sensitivity, specificity, DOR, PLR and NLR of ^18^FDG PET/PET-CT were 0.84 (95% CI = 0.75 to 0.91), 0.98 (95% CI = 0.97 to 0.99), 342 (95% CI = 158 to 740), 54.0 (95% CI = 32.3 to 90.3) and 0.16 (95% CI = 0.10 to 0.26), respectively, and of BS were 0.33 (95% CI = 0.25 to 0.42), 0.98 (95% CI = 0.97 to 0.99), 32 (95% CI = 16 to 62), 21.5 (95% CI = 11.8 to 39.1) and 0.68 (95% CI = 0.59 to 0.77), respectively (Table 3).

### SROC curves

**Figure 3:**
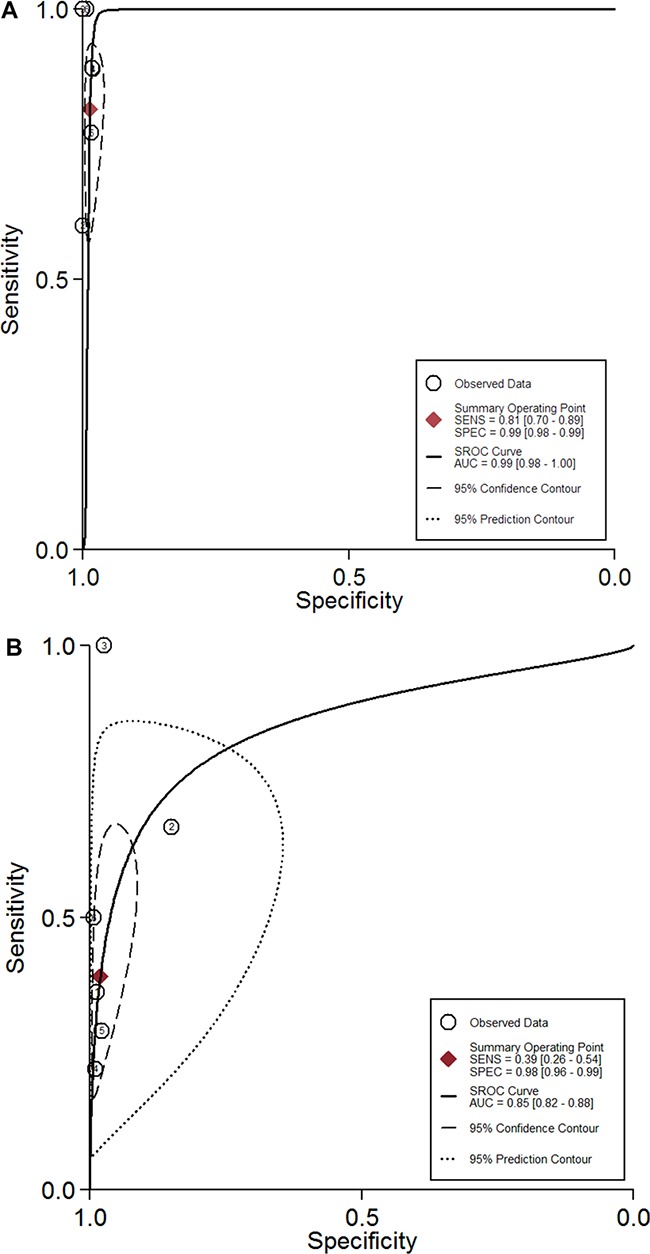
(**A**–**B**) shows the SROC curves of ^18^FDG PET/PET-CT (**A**) and bone scintigraphy (**B**) from all 6 studies.

The SROC curve presents a global summary of test performance, and shows the tradeoff between sensitivity and specificity. The SROC curves of ^18^FDG PET/PET-CT and BS from all 6 studies (1238 patients) were shown in Figure 3A, 3B. Overall weight area under the SROC curves for ^18^FDG PET/PET-CT and BS was 0.98 (95% CI = 0.97 to 0.99) and 0.84 (95% CI = 0.81 to 0.87).

## DISCUSSION

In the past twenty years, ^18^FDG PET or PET-CT entered into clinical usage as a practical imaging technique for distant metastasis staging of nasopharyngeal cancer [1–3, 6–8]. The previous meta-analysis of 8 studies (770 patients) showed that ^18^FDG PET/PET-CT had a sensitivity and a specificity of 0.82 (95% CI = 0.72 to 0.89) and 0.97 (95% CI = 0.95 to 0.98), and conventional imaging procedures (chest radiography, abdominal ultrasonography, and bone scan) had a sensitivity and a specificity of 0.30 (95% CI = 0.19 to 0.44) and 0.97 (95% CI = 0.91 to 0.99) [10]. ^18^FDG PET/PET-CT is more sensitive than conventional imaging procedures for distant metastasis staging in patients with nasopharyngeal cancer. The skeleton is the most frequent distant-site, involving about 60–80% of nasopharyngeal cancer patients with distant metastasis [1–3]. ^99m^Tc-phosphonate-based BS is widely used for detecting bone metastases in nasopharyngeal cancer patients. New imaging techniques such as PET or PET-CT can identify bone metastases at an earlier stage of metastatic tumor growth. But the advantages of ^18^FDG PET/PET-CT over BS are still variable. This meta-analysis showed that ^18^FDG PET/PET-CT was more sensitive than BS (0.81 vs 0.39). The use of PET/PET-CT may provide additional information to BS for diagnosing bone metastatic lesions in nasopharyngeal cancer patients.

The introduction of PET-CT can provide more anatomical details for the PET images. Several studies have demonstrated the diagnostic properties of ^18^FDG PET-CT and BS [2–3, 6–7]. However, the differences for diagnostic accuracy between PET/CT and BS were not clearly delineated. Our meta-analysis showed that ^18^FDG PET-CT was found to have higher sensitivity (0.83 vs 0.46) than BS. The scanner of PET-CT can take the place of BS as a first-line modality for diagnosing bone metastases in nasopharyngeal cancer patients.

Likelihood ratios are considered to be more meaningful for clinical practice. The values of >10 for PLR and < 0.1 for NLR indicate the high accuracy for diagnostic methods [11, 12]. The PLR values of for ^18^FDG PET/PET-CT and BS were 58.6 and 19.9, which were therefore high enough to diagnose bone metastases. But the NLR values for ^18^FDG PET/PET-CT and BS were 0.19 and 0.62, indicating that the negative results of ^18^FDG PET/PET-CT and BS couldn't be used alone as a diagnostic tool to rule out bone metastatic lesions.

There were some limitations in this meta-analysis. First, the publication bias caused by positive results is a major concern, because many studies with nonsignificant or unfavorable results tend to be discarded. In this meta-analysis, publication bias was not performed because of the small number of included studies. Second, there was no consensus for the optimal execution of ^18^FDG PET/PET-CT and BS in all included studies. And the optimal sensitivities and specificities for ^18^FDG PET/PET-CT and BS are still unclear. Third, the included studies did not report sufficient information to separately evaluate the diagnostic value of ^18^FDG PET/PET-CT and BS in early-stage (N_0-1_) and advanced-stage (N_2-3_) patients with nasopharyngeal cancer. Fourth, the gold standard for confirmation of bone metastatic lesions, being histopathologic examination from biopsies, was not obtained from all the lesions in all included studies. However, clinical follow-up results from renewed diagnostic imaging were recorded as a gold standard when histologic confirmation was missing. Fifth, not all included studies had a prospective design. The retrospective studies may have some limitations because the possibility that the imaging interpreters might have known some outcomes of conventional imaging modalities before the interpretation of PET-CT cannot be excluded.

## MATERIALS AND METHODS

### Literature search and study selection

An extensive search was performed to identify relevant articles about the diagnostic efficacy of ^18^FDG PET/PET-CT and BS for detecting bone metastases in nasopharyngeal cancer patients. The MEDLINE and EMBASE databases (last update May 30, 2017) were searched with the following combination of search terms: positron emission tomography, PET, bone metastases, distant metastases, nasopharyngeal cancer, and nasopharyngeal carcinoma. We had no language restrictions for searching relevant studies. References of the retrieved articles were also screened for additional studies.

Studies were eligible for inclusion based on the following criteria: (1) both ^18^FDG PET/PET-CT and BS evaluated bone metastatic cancers in nasopharyngeal cancer patients; 2) histopathology and/or imaging follow-up data were used as the gold standard of diagnosis; (3) the studies were based on a per-patient analysis; and (4) when similar data appeared in more than one article, the article with the most details were chosen. (5) the studies with more than 20 patients were selected for inclusion. Studies were excluded based on the following criteria: (1) only PET/PET-CT or BS was performed; (2) absolute number of true-positive, false-positive, true-negative, and false-negative results were not provided; and (3) the studies were based on a per-lesion analysis.

### Data extraction and quality assessment

Two reviewers (Xu. CH and Zhang. RM) independently extracted the relevant data from each article. And any difference was resolved by consensus. Data was extracted from the included studies, including authors, year of publication, study design, number of patient enrollment, technical characteristics of imaging modalities (PET/PET-CT or BS), and the reference standard. Totals of true positives, false positives, true negatives, and false negatives were also extracted from included studies.

We independently assessed the methodological quality of the included studies using the updated quality assessment tool ‘‘Quality Assessment of Diagnostic Accuracy Studies (QUADAS)-2” [13]. This revised tool allows for more transparent rating of bias and applicability of primary diagnostic accuracy studies. And it may be a considerable improvement over the original assessment tool.

### Statistical analysis

All participants were classified as having positive or negative results of ^18^FDG PET/PET-CT and BS. We used the bivariate model to obtain weighted overall estimates of the sensitivities, specificities, diagnostic odds ratios (DORs), positive/negative likelihood ratios (PLRs/NLRs) as the main outcome measures, and to construct summary receiver operating characteristic (SROC) curves for ^18^FDG PET/PET-CT and BS, respectively [14–15].

All statistical analyses were performed using Stata 11.0 (Stata Corporation, College Station, TX).

## CONCLUSIONS

Compared with BS, ^18^FDG PET/PET-CT has excellent diagnostic performance for the detection of bone metastases in nasopharyngeal cancer patients. ^18^FDG PET/PET-CT may enhance our diagnosis of bone metastases and provide more information for the optimal management of nasopharyngeal cancer patients.
